# Carbon­yl[4-(2,3-dimethyl­phenyl­amino)pent-3-en-2-onato-κ^2^ *N*,*O*](triphenyl­phosphine-κ*P*)rhodium(I)

**DOI:** 10.1107/S1600536809039816

**Published:** 2009-10-07

**Authors:** Gertruida J. S. Venter, Gideon Steyl, Andreas Roodt

**Affiliations:** a Department of Chemistry, University of the Free State, PO Box 339, Bloemfontein 9300, South Africa

## Abstract

In the title compound, [Rh(C_13_H_16_NO)(C_18_H_15_P)(CO)], the coordination geometry of the Rh^I^ atom is square-planar, formed by the coordinating N and O atoms of the bidentate enaminoketonate ligand, one C atom from the carbonyl group and a P atom from triphenyl­phosphine. The complex displays a 0.591 (3):0.409 (3) ratio disorder of the phenyl unit of the monoanionic *N*,*O*-bidentate ligand. Intra­molecular hydrogen bonding is observed between a C—H group of the triphenyl­phosphine unit and the O atom of the enamino­ketonate ligand.

## Related literature

For related derivatives of the 4-phenyl­amino­pent-3-en-2-onate ligand, see: Da Silva *et al.* (1993 ▶); Gordon *et al.* (2002 ▶); Shaheen *et al.* (2006 ▶). For related dicarbonyl rhodium(I) complexes with a bidentate ligand, see: Cornils & Herrmann (1996 ▶); Trzeciak & Ziółkowski (1994 ▶); van Rooy *et al.* (1995 ▶). For related carbonyl rhodium(I) complexes with a phosphine and a bidentate ligand, see: Bonati & Wilkinson (1964 ▶); Damoense *et al.* (1994 ▶); Lamprecht *et al.* (1997 ▶); Leipoldt *et al.* (1978 ▶); Purcell *et al.* (1995 ▶); Varshavsky *et al.* (2001 ▶). For background information, see: Tolman (1977 ▶). 
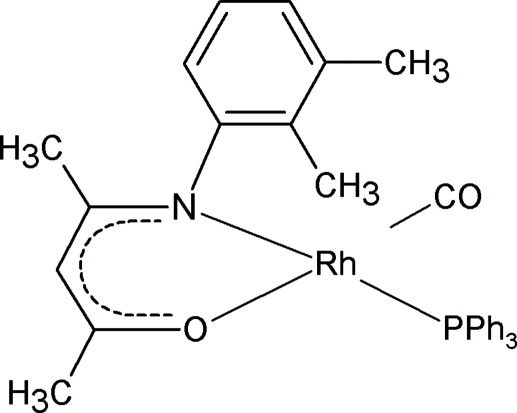


## Experimental

### 

#### Crystal data

[Rh(C_13_H_16_NO)(C_18_H_15_P)(CO)]
*M* *_r_* = 595.46
Monoclinic, 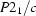

*a* = 14.9077 (3) Å
*b* = 11.6202 (3) Å
*c* = 16.0256 (4) Å
β = 93.521 (1)°
*V* = 2770.88 (11) Å^3^
*Z* = 4
Mo *K*α radiation
μ = 0.70 mm^−1^
*T* = 100 K
0.25 × 0.15 × 0.13 mm

#### Data collection

Bruker X8 APEXII 4K Kappa CCD diffractometer
Absorption correction: multi-scan (*SADABS*; Bruker, 2001 ▶) *T*_min_ = 0.844, *T*_max_ = 0.914
30367 measured reflections
6985 independent reflections
5783 reflections with *I* > 2σ(*I*)
*R*_int_ = 0.040

#### Refinement

*R*[*F* ^2^ > 2σ(*F* ^2^)] = 0.033
*wR*(*F* ^2^) = 0.076
*S* = 1.04
6985 reflections
305 parameters
H-atom parameters constrained
Δρ_max_ = 0.60 e Å^−3^
Δρ_min_ = −0.69 e Å^−3^

### 

Data collection: *APEX2* (Bruker, 2007 ▶); cell refinement: *SAINT-Plus* (Bruker, 2007 ▶); data reduction: *SAINT-Plus*; program(s) used to solve structure: *SIR92* (Altomare *et al.*, 1994 ▶); program(s) used to refine structure: *SHELXL97* (Sheldrick, 2008 ▶); molecular graphics: *DIAMOND* (Brandenburg & Putz, 1999 ▶); software used to prepare material for publication: *WinGX* (Farrugia, 1999 ▶).

## Supplementary Material

Crystal structure: contains datablocks global, I. DOI: 10.1107/S1600536809039816/hy2218sup1.cif

Structure factors: contains datablocks I. DOI: 10.1107/S1600536809039816/hy2218Isup2.hkl

Additional supplementary materials:  crystallographic information; 3D view; checkCIF report

## Figures and Tables

**Table 1:** Hydrogen-bond geometry (Å, °)

*D*—H⋯*A*	*D*—H	H⋯*A*	*D*⋯*A*	*D*—H⋯*A*
C326—H326⋯O12	0.95	2.36	3.177 (3)	143

**Table 2:** Comparative geometrical parameters for similar [Rh(*N*,*O*-bid)(CO)(PPh_3_)] complexes (Å,°)

Parameters	(I)	(II)	(III)
Rh1—N11	2.069 (2)	2.045 (4)	2.045 (3)
Rh1—O12	2.028 (2)	2.044 (3)	2.045 (2)
Rh1—P13	2.2635 (6)	2.275 (1)	2.281 (2)
Rh1—C14	1.807 (2)	1.784 (5)	1.804 (3)
C14—O14	1.152 (3)	1.142 (7)	1.148 (4)
N11⋯O12	2.885 (3)	2.826 (6)	2.841 (3)
N11—Rh1—O12	89.54 (8)	87.4 (1)	87.95 (8)
O12—Rh1—P13	84.97 (5)	89.7 (1)	89.91 (5)
P13—Rh1—C14	91.87 (7)	90.3 (2)	89.48 (9)
N11—Rh1—C14	93.6 (1)	92.6 (2)	92.6 (1)
N11—C2—C4—O12	4.1 (2)	1.2 (4)	1.5 (2)
θ_E_^*a*^	156.39 (3)	156.0 (2)	156.23 (4)

Notes: (I) This work. (II) *N*,*O*-bid = 4-aminopent-3-en-2-onato (Damoense *et al.*, 1994 ▶). (III) *N*,*O*-bid = 4-amino-1,1,1-trifluoropent-3-en-2-onato (Varshavsky *et al.*, 2001 ▶). (*a*) Tolman (1977 ▶)

**Fig. 1.**
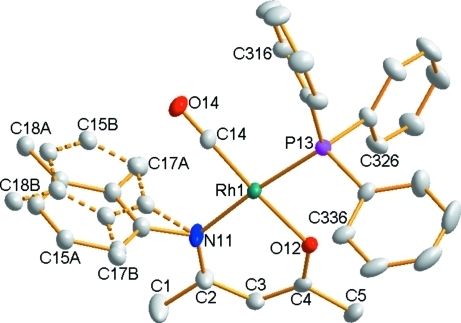
Molecular structure of the title compound. Displacement ellipsoids are drawn at the 50% probability level. H atoms have been omitted for clarity.

**Fig. 2.**
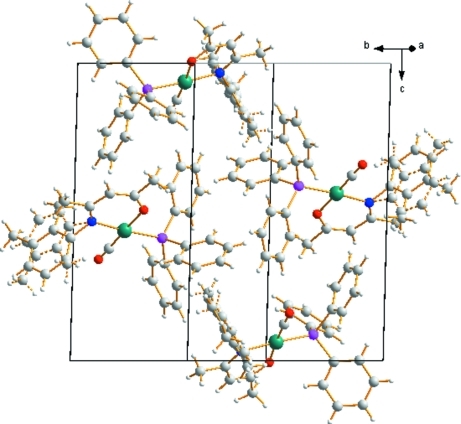
Crystal packing of the title compound.

## supplementary crystallographic information

### Comment

Rhodium(I) dicarbonyl complexes of the type [Rh(*L*,*L*')(CO)_2_] containing chelating mono-anionic bidentate (*L*,*L*') ligands coordinated to rhodium *via* (O,*O*) donor atoms have been studied as catalyst precursors (Cornils & Herrmann, 1996; Trzeciak & Ziółkowski, 1994; van Rooy *et al.*, 1995). In this study the investigation of these β-diketonato complexes is followed by complexes containing bidentate β-enaminoketonato ligands such as 4-(phenylamino)pent-3-en-2-onato (Phony) (Shaheen *et al.*, 2006) coordinated to rhodium *via* (N,*O*) donor atoms. Dicarbonyl complexes of the [Rh(N,*O*-bid)(CO)_2_]-type (Varshavsky *et al.*, 2001) react with phosphorus ligands to form [Rh(N,*O*-bid)(CO)(P*Z*_3_)] complexes (Damoense *et al.*, 1994; Varshavsky *et al.*, 2001). According to Bonati & Wilkinson (1964), only one CO group will be substituted by triphenylphosphine, with the product being one of two possible isomers. Since N atom has a larger *trans*-influence than O atom, the CO group *trans* to the N atom will be substituted. This is evident in the title compound (Fig. 1), where [Rh(2,3-diMe-Phony)(CO)(PPh_3_)] is formed by the substitution of the carbonyl ligand in the dicarbonyl rhodium(I) complex [Rh(2,3-diMe-Phony)(CO)_2_] by PPh_3_.

Bond distances involving Rh atom in the title complex differ significantly from the distances in related complexes (Table 2). The Rh—N bond distance in the title complex is longer than those in similar complexes while the Rh—O bond distance is shorter. This is due to the steric influence of the phenyl group connected to N atom in the title compound, as opposed to H atom in the related complexes. The Rh—C and the carbonyl C—O bond distances do not differ substantially from the distances in the related complexes (Table 2). The N—Rh—O bite angle is slightly larger than those observed in similar complexes found in literature. The effective cone angle, θ_E_ (Tolman, 1977), of 156.39 (3)° is similar to the angles in the related compounds. The title complex displays a disorder of the phenyl ring in a 59:41% ratio.

### Experimental

To a 5 ml acetone solution of [Rh(2,3-diMe-Phony)(CO)_2_] (0.0204 g, 56.48 mmol) was added PPh_3_ (0.0151 g, 57.57 mmol) resulting in the immediate evolution of gas. Crystallization from acetone produced yellow crystals in quantitative yield (0.0334 g). IR (KBr): ν_CO_ 1966.93 s (cm^-1^).

### Refinement

The methyl and aromatic H atoms were placed in geometrically idealized positions and constrained to ride on their parent atoms, with C—H = 0.98 and 0.95 Å and *U*_iso_(H) = 1.5*U*_eq_(C) and 1.2*U*_eq_(C), respectively. The methyl groups were generated to fit the difference electron density and the groups were then refined as rigid rotors.

### Crystal data

**Table:**

[Rh(C_13_H_16_NO)(C_18_H_15_P)(CO)]	*F*(000) = 1224
*M**_r_* = 595.46	*D*_x_ = 1.427 Mg m^−^^3^
Monoclinic, *P*2_1_/*c*	Mo *K*α radiation, λ = 0.71073 Å
Hall symbol: -P 2ybc	Cell parameters from 8984 reflections
*a* = 14.9077 (3) Å	θ = 2.2–28.4°
*b* = 11.6202 (3) Å	µ = 0.70 mm^−^^1^
*c* = 16.0256 (4) Å	*T* = 100 K
β = 93.521 (1)°	Cuboid, yellow
*V* = 2770.88 (11) Å^3^	0.25 × 0.15 × 0.13 mm
*Z* = 4	

### Data collection

**Table:**

Bruker X8 APEXII 4K Kappa CCD diffractometer	6985 independent reflections
Radiation source: fine-focus sealed tube	5783 reflections with *I* > 2σ(*I*)
graphite	*R*_int_ = 0.040
ω and φ scans	θ_max_ = 28.5°, θ_min_ = 1.4°
Absorption correction: multi-scan (*SADABS*; Bruker, 2001)	*h* = −19→19
*T*_min_ = 0.844, *T*_max_ = 0.914	*k* = −15→14
30367 measured reflections	*l* = −21→18

### Refinement

**Table:**

Refinement on *F*^2^	Primary atom site location: structure-invariant direct methods
Least-squares matrix: full	Secondary atom site location: difference Fourier map
*R*[*F*^2^ > 2σ(*F*^2^)] = 0.033	Hydrogen site location: inferred from neighbouring sites
*wR*(*F*^2^) = 0.076	H-atom parameters constrained
*S* = 1.04	*w* = 1/[σ^2^(*F*_o_^2^) + (0.0235*P*)^2^ + 2.8578*P*] where *P* = (*F*_o_^2^ + 2*F*_c_^2^)/3
6985 reflections	(Δ/σ)_max_ = 0.005
305 parameters	Δρ_max_ = 0.60 e Å^−^^3^
0 restraints	Δρ_min_ = −0.69 e Å^−^^3^

### Special details

**Table:**

Experimental. The intensity data was collected on a Bruker X8 APEXII 4 K Kappa CCD diffractometer using an exposure time of 60 s/frame. A total of 1033 frames were collected with a frame width of 0.5° covering up to θ = 28.41° with 99.4% completeness accomplished.

### Fractional atomic coordinates and isotropic or equivalent isotropic displacement parameters (Å^2^)

**Table:**

	*x*	*y*	*z*	*U*_iso_*/*U*_eq_	Occ. (<1)
Rh1	0.258727 (11)	0.605541 (15)	1.062396 (10)	0.01882 (6)	
N11	0.26580 (15)	0.43296 (18)	1.03172 (15)	0.0361 (5)	
O12	0.36554 (10)	0.63702 (14)	0.99328 (9)	0.0221 (3)	
O14	0.09801 (11)	0.57318 (15)	1.15945 (10)	0.0285 (4)	
P13	0.26175 (4)	0.79721 (5)	1.08760 (3)	0.01818 (12)	
C1	0.3204 (2)	0.2591 (2)	0.9641 (2)	0.0576 (10)	
H1A	0.3099	0.218	1.0161	0.086*	
H1B	0.3782	0.2352	0.9439	0.086*	
H1C	0.2722	0.2409	0.9219	0.086*	
C2	0.32179 (18)	0.3869 (2)	0.98045 (17)	0.0325 (6)	
C3	0.38454 (16)	0.4508 (2)	0.93713 (15)	0.0267 (5)	
H3	0.4176	0.4093	0.8982	0.032*	
C4	0.40317 (14)	0.5655 (2)	0.94473 (14)	0.0222 (5)	
C5	0.47221 (16)	0.6203 (2)	0.89254 (16)	0.0318 (6)	
H5A	0.4419	0.6684	0.8492	0.048*	
H5B	0.5068	0.5601	0.8662	0.048*	
H5C	0.5129	0.668	0.9283	0.048*	
C11A	0.22412 (17)	0.3512 (2)	1.08695 (16)	0.0249 (5)	0.591 (3)
C12A	0.13769 (16)	0.3169 (2)	1.06046 (13)	0.0249 (5)	0.591 (3)
C13A	0.09120 (13)	0.2400 (2)	1.10833 (15)	0.0249 (5)	0.591 (3)
C14A	0.13113 (16)	0.1974 (2)	1.18269 (14)	0.0249 (5)	0.591 (3)
H14A	0.0994	0.1449	1.2154	0.03*	0.591 (3)
C15A	0.21756 (17)	0.2318 (2)	1.20919 (14)	0.0249 (5)	0.591 (3)
H15A	0.2449	0.2027	1.26	0.03*	0.591 (3)
C16A	0.26405 (14)	0.3086 (3)	1.16132 (17)	0.0249 (5)	0.591 (3)
H16A	0.3231	0.3321	1.1794	0.03*	0.591 (3)
C18A	−0.0011 (3)	0.1950 (4)	1.0797 (2)	0.0249 (5)	0.591 (3)
H18A	0.0043	0.1423	1.0325	0.037*	0.591 (3)
H18B	−0.0401	0.2596	1.0622	0.037*	0.591 (3)
H18C	−0.0271	0.1539	1.1258	0.037*	0.591 (3)
C17A	0.0953 (3)	0.3664 (4)	0.9799 (3)	0.0249 (5)	0.591 (3)
H17A	0.0902	0.4501	0.9853	0.037*	0.591 (3)
H17B	0.0354	0.333	0.9688	0.037*	0.591 (3)
H17C	0.1329	0.348	0.9337	0.037*	0.591 (3)
C11B	0.1803 (2)	0.3671 (3)	1.0474 (2)	0.0243 (7)	0.409 (3)
C12B	0.18861 (19)	0.3040 (3)	1.1212 (2)	0.0243 (7)	0.409 (3)
C13B	0.1158 (2)	0.2418 (3)	1.14741 (19)	0.0243 (7)	0.409 (3)
C14B	0.0346 (2)	0.2428 (3)	1.0998 (2)	0.0243 (7)	0.409 (3)
H14B	−0.0152	0.2003	1.1178	0.029*	0.409 (3)
C15B	0.0263 (2)	0.3059 (3)	1.0261 (2)	0.0243 (7)	0.409 (3)
H15B	−0.0292	0.3065	0.9936	0.029*	0.409 (3)
C16B	0.0991 (3)	0.3680 (3)	0.99988 (19)	0.0243 (7)	0.409 (3)
H16B	0.0934	0.4111	0.9495	0.029*	0.409 (3)
C17B	0.2760 (4)	0.3064 (6)	1.1737 (4)	0.0243 (7)	0.409 (3)
H17D	0.3093	0.2351	1.165	0.036*	0.409 (3)
H17E	0.2636	0.3132	1.2328	0.036*	0.409 (3)
H17F	0.3119	0.3724	1.1574	0.036*	0.409 (3)
C18B	0.1219 (4)	0.1738 (5)	1.2287 (3)	0.0243 (7)	0.409 (3)
H18D	0.0624	0.144	1.2399	0.036*	0.409 (3)
H18E	0.1436	0.2241	1.2747	0.036*	0.409 (3)
H18F	0.1638	0.1094	1.2238	0.036*	0.409 (3)
C14	0.16135 (15)	0.58652 (19)	1.12283 (14)	0.0215 (5)	
C311	0.19823 (15)	0.8522 (2)	1.17253 (14)	0.0212 (5)	
C312	0.23991 (16)	0.8923 (2)	1.24725 (15)	0.0261 (5)	
H312	0.3036	0.898	1.2531	0.031*	
C313	0.18891 (18)	0.9239 (2)	1.31310 (16)	0.0336 (6)	
H313	0.2177	0.9501	1.3642	0.04*	
C314	0.09599 (18)	0.9173 (2)	1.30437 (17)	0.0372 (6)	
H314	0.0611	0.9395	1.3493	0.045*	
C315	0.05421 (17)	0.8787 (2)	1.23056 (18)	0.0364 (6)	
H315	−0.0095	0.8745	1.2248	0.044*	
C316	0.10419 (16)	0.8460 (2)	1.16490 (16)	0.0289 (5)	
H316	0.0748	0.8192	1.1143	0.035*	
C321	0.22426 (16)	0.8831 (2)	0.99645 (14)	0.0239 (5)	
C322	0.17703 (18)	0.9858 (2)	1.00150 (15)	0.0329 (6)	
H322	0.1615	1.014	1.0544	0.04*	
C323	0.1525 (2)	1.0472 (2)	0.92915 (17)	0.0409 (7)	
H323	0.1194	1.1167	0.9329	0.049*	
C324	0.1756 (2)	1.0084 (2)	0.85193 (16)	0.0383 (6)	
H324	0.1591	1.0511	0.8028	0.046*	
C325	0.22301 (19)	0.9068 (2)	0.84678 (16)	0.0362 (6)	
H325	0.2393	0.8796	0.7939	0.043*	
C326	0.24695 (16)	0.8442 (2)	0.91864 (14)	0.0280 (5)	
H326	0.2791	0.7741	0.9144	0.034*	
C331	0.37540 (15)	0.8504 (2)	1.11391 (13)	0.0224 (5)	
C332	0.40227 (19)	0.9603 (2)	1.09345 (18)	0.0389 (7)	
H332	0.3623	1.0096	1.0618	0.047*	
C333	0.4886 (2)	0.9988 (3)	1.1196 (2)	0.0510 (8)	
H333	0.5073	1.074	1.1053	0.061*	
C334	0.54615 (18)	0.9282 (3)	1.16566 (18)	0.0431 (8)	
H334	0.6043	0.955	1.1839	0.052*	
C335	0.52026 (16)	0.8193 (3)	1.18548 (16)	0.0353 (6)	
H335	0.5605	0.7705	1.2172	0.042*	
C336	0.43533 (15)	0.7797 (2)	1.15942 (14)	0.0264 (5)	
H336	0.4181	0.7035	1.1729	0.032*	

### Atomic displacement parameters (Å^2^)

**Table:**

	*U*^11^	*U*^22^	*U*^33^	*U*^12^	*U*^13^	*U*^23^
Rh1	0.01877 (9)	0.01664 (9)	0.02180 (9)	−0.00199 (7)	0.00727 (6)	−0.00079 (7)
N11	0.0465 (13)	0.0177 (10)	0.0478 (13)	−0.0072 (9)	0.0327 (11)	−0.0047 (9)
O12	0.0197 (8)	0.0216 (8)	0.0256 (8)	0.0005 (6)	0.0075 (6)	0.0035 (6)
O14	0.0241 (9)	0.0315 (10)	0.0309 (9)	−0.0077 (7)	0.0108 (7)	−0.0045 (7)
P13	0.0172 (3)	0.0182 (3)	0.0191 (3)	−0.0007 (2)	0.0002 (2)	−0.0006 (2)
C1	0.083 (2)	0.0238 (15)	0.073 (2)	−0.0050 (15)	0.055 (2)	−0.0111 (15)
C2	0.0384 (14)	0.0220 (13)	0.0393 (14)	−0.0005 (11)	0.0201 (12)	−0.0043 (11)
C3	0.0271 (12)	0.0248 (13)	0.0297 (12)	0.0038 (10)	0.0134 (10)	−0.0004 (10)
C4	0.0169 (10)	0.0277 (12)	0.0223 (11)	0.0033 (9)	0.0038 (9)	0.0054 (9)
C5	0.0247 (12)	0.0372 (15)	0.0349 (13)	−0.0004 (11)	0.0128 (10)	0.0068 (11)
C11A	0.0258 (8)	0.0232 (8)	0.0259 (8)	−0.0024 (6)	0.0018 (6)	0.0011 (6)
C12A	0.0258 (8)	0.0232 (8)	0.0259 (8)	−0.0024 (6)	0.0018 (6)	0.0011 (6)
C13A	0.0258 (8)	0.0232 (8)	0.0259 (8)	−0.0024 (6)	0.0018 (6)	0.0011 (6)
C14A	0.0258 (8)	0.0232 (8)	0.0259 (8)	−0.0024 (6)	0.0018 (6)	0.0011 (6)
C15A	0.0258 (8)	0.0232 (8)	0.0259 (8)	−0.0024 (6)	0.0018 (6)	0.0011 (6)
C16A	0.0258 (8)	0.0232 (8)	0.0259 (8)	−0.0024 (6)	0.0018 (6)	0.0011 (6)
C18A	0.0258 (8)	0.0232 (8)	0.0259 (8)	−0.0024 (6)	0.0018 (6)	0.0011 (6)
C17A	0.0258 (8)	0.0232 (8)	0.0259 (8)	−0.0024 (6)	0.0018 (6)	0.0011 (6)
C11B	0.0281 (12)	0.0204 (11)	0.0246 (11)	0.0016 (9)	0.0036 (9)	0.0003 (8)
C12B	0.0281 (12)	0.0204 (11)	0.0246 (11)	0.0016 (9)	0.0036 (9)	0.0003 (8)
C13B	0.0281 (12)	0.0204 (11)	0.0246 (11)	0.0016 (9)	0.0036 (9)	0.0003 (8)
C14B	0.0281 (12)	0.0204 (11)	0.0246 (11)	0.0016 (9)	0.0036 (9)	0.0003 (8)
C15B	0.0281 (12)	0.0204 (11)	0.0246 (11)	0.0016 (9)	0.0036 (9)	0.0003 (8)
C16B	0.0281 (12)	0.0204 (11)	0.0246 (11)	0.0016 (9)	0.0036 (9)	0.0003 (8)
C17B	0.0281 (12)	0.0204 (11)	0.0246 (11)	0.0016 (9)	0.0036 (9)	0.0003 (8)
C18B	0.0281 (12)	0.0204 (11)	0.0246 (11)	0.0016 (9)	0.0036 (9)	0.0003 (8)
C14	0.0238 (11)	0.0192 (12)	0.0216 (11)	−0.0045 (9)	0.0027 (9)	−0.0031 (9)
C311	0.0201 (11)	0.0193 (11)	0.0242 (11)	0.0002 (9)	0.0009 (9)	−0.0020 (9)
C312	0.0232 (12)	0.0279 (13)	0.0267 (12)	0.0035 (10)	−0.0017 (9)	−0.0048 (10)
C313	0.0363 (14)	0.0400 (16)	0.0242 (12)	0.0017 (12)	−0.0009 (11)	−0.0116 (11)
C314	0.0320 (14)	0.0462 (17)	0.0345 (14)	0.0044 (12)	0.0106 (11)	−0.0135 (12)
C315	0.0201 (12)	0.0439 (17)	0.0459 (16)	−0.0004 (11)	0.0072 (11)	−0.0112 (13)
C316	0.0235 (12)	0.0312 (14)	0.0319 (13)	−0.0013 (10)	0.0007 (10)	−0.0085 (11)
C321	0.0280 (12)	0.0199 (12)	0.0231 (11)	−0.0001 (10)	−0.0034 (9)	−0.0003 (9)
C322	0.0483 (16)	0.0230 (13)	0.0267 (12)	0.0066 (12)	−0.0043 (11)	−0.0046 (10)
C323	0.0585 (19)	0.0243 (14)	0.0386 (15)	0.0148 (13)	−0.0064 (14)	0.0002 (11)
C324	0.0529 (18)	0.0312 (15)	0.0298 (13)	0.0095 (13)	−0.0065 (12)	0.0074 (11)
C325	0.0469 (16)	0.0383 (16)	0.0230 (12)	0.0103 (13)	−0.0008 (11)	0.0022 (11)
C326	0.0340 (14)	0.0248 (13)	0.0246 (12)	0.0081 (10)	−0.0023 (10)	0.0009 (10)
C331	0.0200 (11)	0.0286 (12)	0.0190 (10)	−0.0059 (9)	0.0035 (9)	−0.0027 (9)
C332	0.0405 (16)	0.0334 (16)	0.0423 (15)	−0.0137 (12)	−0.0020 (13)	0.0053 (12)
C333	0.0503 (19)	0.0486 (19)	0.0550 (19)	−0.0328 (16)	0.0110 (15)	−0.0046 (16)
C334	0.0240 (13)	0.067 (2)	0.0387 (15)	−0.0154 (14)	0.0085 (12)	−0.0198 (15)
C335	0.0194 (12)	0.0592 (19)	0.0276 (12)	0.0007 (12)	0.0031 (10)	−0.0165 (12)
C336	0.0212 (11)	0.0368 (14)	0.0215 (11)	−0.0014 (10)	0.0050 (9)	−0.0095 (10)

### Geometric parameters (Å, °)

**Table:**

Rh1—C14	1.807 (2)	C13B—C18B	1.522 (6)
Rh1—O12	2.0280 (15)	C14B—C15B	1.39
Rh1—N11	2.069 (2)	C14B—H14B	0.95
Rh1—P13	2.2635 (6)	C15B—C16B	1.39
N11—C2	1.320 (3)	C15B—H15B	0.95
N11—C11A	1.463 (3)	C16B—H16B	0.95
N11—C11B	1.522 (3)	C17B—H17D	0.98
O12—C4	1.290 (3)	C17B—H17E	0.98
O14—C14	1.152 (3)	C17B—H17F	0.98
P13—C311	1.821 (2)	C18B—H18D	0.98
P13—C331	1.828 (2)	C18B—H18E	0.98
P13—C321	1.828 (2)	C18B—H18F	0.98
C1—C2	1.508 (4)	C311—C312	1.395 (3)
C1—H1A	0.98	C311—C316	1.401 (3)
C1—H1B	0.98	C312—C313	1.388 (3)
C1—H1C	0.98	C312—H312	0.95
C2—C3	1.410 (3)	C313—C314	1.386 (4)
C3—C4	1.366 (3)	C313—H313	0.95
C3—H3	0.95	C314—C315	1.378 (4)
C4—C5	1.507 (3)	C314—H314	0.95
C5—H5A	0.98	C315—C316	1.379 (3)
C5—H5B	0.98	C315—H315	0.95
C5—H5C	0.98	C316—H316	0.95
C11A—C12A	1.39	C321—C326	1.388 (3)
C11A—C16A	1.39	C321—C322	1.390 (3)
C12A—C13A	1.39	C322—C323	1.391 (4)
C12A—C17A	1.515 (5)	C322—H322	0.95
C13A—C14A	1.39	C323—C324	1.381 (4)
C13A—C18A	1.516 (4)	C323—H323	0.95
C14A—C15A	1.39	C324—C325	1.380 (4)
C14A—H14A	0.95	C324—H324	0.95
C15A—C16A	1.39	C325—C326	1.390 (3)
C15A—H15A	0.95	C325—H325	0.95
C16A—H16A	0.95	C326—H326	0.95
C18A—H18A	0.98	C331—C332	1.384 (4)
C18A—H18B	0.98	C331—C336	1.388 (3)
C18A—H18C	0.98	C332—C333	1.403 (4)
C17A—H17A	0.98	C332—H332	0.95
C17A—H17B	0.98	C333—C334	1.369 (5)
C17A—H17C	0.98	C333—H333	0.95
C11B—C12B	1.39	C334—C335	1.366 (4)
C11B—C16B	1.39	C334—H334	0.95
C12B—C13B	1.39	C335—C336	1.387 (3)
C12B—C17B	1.506 (7)	C335—H335	0.95
C13B—C14B	1.39	C336—H336	0.95
			
C14—Rh1—O12	176.48 (9)	C14B—C15B—H15B	120
C14—Rh1—N11	93.62 (9)	C15B—C16B—C11B	120
O12—Rh1—N11	89.53 (7)	C15B—C16B—H16B	120
C14—Rh1—P13	91.86 (7)	C11B—C16B—H16B	120
O12—Rh1—P13	84.97 (5)	C12B—C17B—H17D	109.5
N11—Rh1—P13	174.49 (6)	C12B—C17B—H17E	109.5
C2—N11—C11A	114.9 (2)	H17D—C17B—H17E	109.5
C2—N11—C11B	117.8 (2)	C12B—C17B—H17F	109.5
C2—N11—Rh1	125.77 (17)	H17D—C17B—H17F	109.5
C11A—N11—Rh1	117.14 (17)	H17E—C17B—H17F	109.5
C11B—N11—Rh1	113.14 (19)	C13B—C18B—H18D	109.5
C4—O12—Rh1	126.75 (15)	C13B—C18B—H18E	109.5
C311—P13—C331	103.07 (10)	H18D—C18B—H18E	109.5
C311—P13—C321	104.96 (11)	C13B—C18B—H18F	109.5
C331—P13—C321	103.48 (11)	H18D—C18B—H18F	109.5
C311—P13—Rh1	118.23 (8)	H18E—C18B—H18F	109.5
C331—P13—Rh1	112.44 (8)	O14—C14—Rh1	178.1 (2)
C321—P13—Rh1	113.14 (8)	C312—C311—C316	118.8 (2)
C2—C1—H1A	109.5	C312—C311—P13	122.29 (17)
C2—C1—H1B	109.5	C316—C311—P13	118.70 (17)
H1A—C1—H1B	109.5	C313—C312—C311	120.4 (2)
C2—C1—H1C	109.5	C313—C312—H312	119.8
H1A—C1—H1C	109.5	C311—C312—H312	119.8
H1B—C1—H1C	109.5	C314—C313—C312	120.0 (2)
N11—C2—C3	123.8 (2)	C314—C313—H313	120
N11—C2—C1	120.3 (2)	C312—C313—H313	120
C3—C2—C1	115.8 (2)	C315—C314—C313	120.1 (2)
C4—C3—C2	127.4 (2)	C315—C314—H314	120
C4—C3—H3	116.3	C313—C314—H314	120
C2—C3—H3	116.3	C314—C315—C316	120.5 (2)
O12—C4—C3	126.1 (2)	C314—C315—H315	119.7
O12—C4—C5	113.6 (2)	C316—C315—H315	119.7
C3—C4—C5	120.3 (2)	C315—C316—C311	120.2 (2)
C4—C5—H5A	109.5	C315—C316—H316	119.9
C4—C5—H5B	109.5	C311—C316—H316	119.9
H5A—C5—H5B	109.5	C326—C321—C322	119.0 (2)
C4—C5—H5C	109.5	C326—C321—P13	117.40 (18)
H5A—C5—H5C	109.5	C322—C321—P13	123.59 (18)
H5B—C5—H5C	109.5	C321—C322—C323	120.0 (2)
C12A—C11A—C16A	120	C321—C322—H322	120
C12A—C11A—N11	114.94 (19)	C323—C322—H322	120
C16A—C11A—N11	125.06 (19)	C324—C323—C322	120.8 (2)
C11A—C12A—C13A	120	C324—C323—H323	119.6
C11A—C12A—C17A	118.8 (2)	C322—C323—H323	119.6
C13A—C12A—C17A	121.2 (2)	C325—C324—C323	119.3 (2)
C12A—C13A—C14A	120	C325—C324—H324	120.3
C12A—C13A—C18A	121.9 (2)	C323—C324—H324	120.3
C14A—C13A—C18A	118.0 (2)	C324—C325—C326	120.3 (2)
C15A—C14A—C13A	120	C324—C325—H325	119.9
C15A—C14A—H14A	120	C326—C325—H325	119.9
C13A—C14A—H14A	120	C321—C326—C325	120.6 (2)
C14A—C15A—C16A	120	C321—C326—H326	119.7
C14A—C15A—H15A	120	C325—C326—H326	119.7
C16A—C15A—H15A	120	C332—C331—C336	119.0 (2)
C15A—C16A—C11A	120	C332—C331—P13	122.3 (2)
C15A—C16A—H16A	120	C336—C331—P13	118.61 (18)
C11A—C16A—H16A	120	C331—C332—C333	119.8 (3)
C12B—C11B—C16B	120	C331—C332—H332	120.1
C12B—C11B—N11	112.0 (2)	C333—C332—H332	120.1
C16B—C11B—N11	128.0 (2)	C334—C333—C332	120.2 (3)
C11B—C12B—C13B	120	C334—C333—H333	119.9
C11B—C12B—C17B	119.6 (3)	C332—C333—H333	119.9
C13B—C12B—C17B	120.4 (3)	C335—C334—C333	120.3 (3)
C14B—C13B—C12B	120	C335—C334—H334	119.9
C14B—C13B—C18B	118.6 (3)	C333—C334—H334	119.9
C12B—C13B—C18B	121.4 (3)	C334—C335—C336	120.2 (3)
C13B—C14B—C15B	120	C334—C335—H335	119.9
C13B—C14B—H14B	120	C336—C335—H335	119.9
C15B—C14B—H14B	120	C335—C336—C331	120.5 (3)
C16B—C15B—C14B	120	C335—C336—H336	119.7
C16B—C15B—H15B	120	C331—C336—H336	119.7
			
C14—Rh1—N11—C2	174.7 (3)	C16B—C11B—C12B—C17B	−178.1 (4)
O12—Rh1—N11—C2	−3.8 (3)	N11—C11B—C12B—C17B	−0.6 (4)
C14—Rh1—N11—C11A	−23.0 (2)	C11B—C12B—C13B—C14B	0
O12—Rh1—N11—C11A	158.5 (2)	C17B—C12B—C13B—C14B	178.1 (4)
C14—Rh1—N11—C11B	15.6 (2)	C11B—C12B—C13B—C18B	−178.8 (4)
O12—Rh1—N11—C11B	−162.8 (2)	C17B—C12B—C13B—C18B	−0.7 (5)
N11—Rh1—O12—C4	8.13 (19)	C12B—C13B—C14B—C15B	0
P13—Rh1—O12—C4	−171.45 (18)	C18B—C13B—C14B—C15B	178.8 (4)
C14—Rh1—P13—C311	16.41 (11)	C13B—C14B—C15B—C16B	0
O12—Rh1—P13—C311	−165.13 (10)	C14B—C15B—C16B—C11B	0
C14—Rh1—P13—C331	136.38 (11)	C12B—C11B—C16B—C15B	0
O12—Rh1—P13—C331	−45.16 (9)	N11—C11B—C16B—C15B	−177.0 (4)
C14—Rh1—P13—C321	−106.82 (11)	C331—P13—C311—C312	−16.6 (2)
O12—Rh1—P13—C321	71.63 (10)	C321—P13—C311—C312	−124.6 (2)
C11A—N11—C2—C3	−164.1 (3)	Rh1—P13—C311—C312	108.17 (19)
C11B—N11—C2—C3	156.7 (3)	C331—P13—C311—C316	168.3 (2)
Rh1—N11—C2—C3	−1.5 (4)	C321—P13—C311—C316	60.2 (2)
C11A—N11—C2—C1	16.8 (4)	Rh1—P13—C311—C316	−67.0 (2)
C11B—N11—C2—C1	−22.4 (4)	C316—C311—C312—C313	0.9 (4)
Rh1—N11—C2—C1	179.4 (2)	P13—C311—C312—C313	−174.3 (2)
N11—C2—C3—C4	5.4 (5)	C311—C312—C313—C314	−0.9 (4)
C1—C2—C3—C4	−175.5 (3)	C312—C313—C314—C315	0.4 (4)
Rh1—O12—C4—C3	−7.5 (3)	C313—C314—C315—C316	0.1 (5)
Rh1—O12—C4—C5	171.45 (15)	C314—C315—C316—C311	−0.2 (4)
C2—C3—C4—O12	−0.5 (4)	C312—C311—C316—C315	−0.3 (4)
C2—C3—C4—C5	−179.4 (3)	P13—C311—C316—C315	175.1 (2)
C2—N11—C11A—C12A	−98.8 (3)	C311—P13—C321—C326	−166.46 (19)
C11B—N11—C11A—C12A	4.8 (3)	C331—P13—C321—C326	85.8 (2)
Rh1—N11—C11A—C12A	97.0 (2)	Rh1—P13—C321—C326	−36.2 (2)
C2—N11—C11A—C16A	81.7 (3)	C311—P13—C321—C322	15.4 (2)
C11B—N11—C11A—C16A	−174.7 (4)	C331—P13—C321—C322	−92.3 (2)
Rh1—N11—C11A—C16A	−82.5 (2)	Rh1—P13—C321—C322	145.7 (2)
C16A—C11A—C12A—C13A	0	C326—C321—C322—C323	0.6 (4)
N11—C11A—C12A—C13A	−179.5 (3)	P13—C321—C322—C323	178.7 (2)
C16A—C11A—C12A—C17A	178.9 (3)	C321—C322—C323—C324	−1.0 (4)
N11—C11A—C12A—C17A	−0.6 (3)	C322—C323—C324—C325	0.6 (5)
C11A—C12A—C13A—C14A	0	C323—C324—C325—C326	0.2 (5)
C17A—C12A—C13A—C14A	−178.9 (3)	C322—C321—C326—C325	0.1 (4)
C11A—C12A—C13A—C18A	−176.5 (3)	P13—C321—C326—C325	−178.1 (2)
C17A—C12A—C13A—C18A	4.6 (4)	C324—C325—C326—C321	−0.5 (4)
C12A—C13A—C14A—C15A	0	C311—P13—C331—C332	−83.3 (2)
C18A—C13A—C14A—C15A	176.7 (3)	C321—P13—C331—C332	25.9 (2)
C13A—C14A—C15A—C16A	0	Rh1—P13—C331—C332	148.32 (19)
C14A—C15A—C16A—C11A	0	C311—P13—C331—C336	93.63 (19)
C12A—C11A—C16A—C15A	0	C321—P13—C331—C336	−157.22 (18)
N11—C11A—C16A—C15A	179.5 (3)	Rh1—P13—C331—C336	−34.78 (19)
C2—N11—C11B—C12B	98.0 (3)	C336—C331—C332—C333	−0.7 (4)
C11A—N11—C11B—C12B	3.6 (3)	P13—C331—C332—C333	176.2 (2)
Rh1—N11—C11B—C12B	−101.2 (2)	C331—C332—C333—C334	−0.4 (4)
C2—N11—C11B—C16B	−84.8 (3)	C332—C333—C334—C335	0.9 (4)
C11A—N11—C11B—C16B	−179.2 (5)	C333—C334—C335—C336	−0.3 (4)
Rh1—N11—C11B—C16B	76.1 (3)	C334—C335—C336—C331	−0.8 (3)
C16B—C11B—C12B—C13B	0	C332—C331—C336—C335	1.3 (3)
N11—C11B—C12B—C13B	177.5 (3)	P13—C331—C336—C335	−175.74 (17)

### Hydrogen-bond geometry (Å, °)

**Table:**

*D*—H···*A*	*D*—H	H···*A*	*D*···*A*	*D*—H···*A*
C326—H326···O12	0.95	2.36	3.177 (3)	143

### Table 2 Comparative geometrical parameters for similar [Rh(N,O-bid)(CO)(PPh_3_)] complexes (Å,°).

**Table:**

Parameters	(I)	(II)	(III)
Rh1—N11	2.069 (2)	2.045 (4)	2.045 (3)
Rh1—O12	2.028 (2)	2.044 (3)	2.045 (2)
Rh1—P13	2.2635 (6)	2.275 (1)	2.281 (2)
Rh1—C14	1.807 (2)	1.784 (5)	1.804 (3)
C14—O14	1.152 (3)	1.142 (7)	1.148 (4)
N11···O12	2.885 (3)	2.826 (6)	2.841 (3)
N11—Rh1—O12	89.54 (8)	87.4 (1)	87.95 (8)
O12—Rh1—P13	84.97 (5)	89.7 (1)	89.91 (5)
P13—Rh1—C14	91.87 (7)	90.3 (2)	89.48 (9)
N11—Rh1—C14	93.6 (1)	92.6 (2)	92.6 (1)
N11—C2—C4—O12	4.1 (2)	1.2 (4)	1.5 (2)
θ_E_ (Tolman, 1977)	156.39 (3)	156.0 (2)	156.23 (4)

(I) This work. (II) N,O-bid = 4-aminopent-3-en-2-onato (Damoense *et al.*, 1994). (III) N,O-bid = 4-amino-1,1,1-trifluoropent-3-en-2-onato (Varshavsky *et al.*, 2001).
